# *De Novo* Transcriptome Sequencing of the *Octopus vulgaris* Hemocytes Using Illumina RNA-Seq Technology: Response to the Infection by the Gastrointestinal Parasite *Aggregata octopiana*

**DOI:** 10.1371/journal.pone.0107873

**Published:** 2014-10-16

**Authors:** Sheila Castellanos-Martínez, David Arteta, Susana Catarino, Camino Gestal

**Affiliations:** 1 Departamento de Biotecnología y Acuicultura. Instituto de Investigaciones Marinas, Consejo Superior de Investigaciones Científicas, Vigo, Spain; 2 PROGENIKA Biopharma. A Grifols Company. Parque tecnológico de Bizkaia. Derio, Bizkaia, Spain; George Washington University School of Medicine and Health Sciences, United States of America

## Abstract

**Background:**

*Octopus vulgaris* is a highly valuable species of great commercial interest and excellent candidate for aquaculture diversification; however, the octopus’ well-being is impaired by pathogens, of which the gastrointestinal coccidian parasite *Aggregata octopiana* is one of the most important. The knowledge of the molecular mechanisms of the immune response in cephalopods, especially in octopus is scarce. The transcriptome of the hemocytes of *O. vulgaris* was *de novo* sequenced using the high-throughput paired-end Illumina technology to identify genes involved in immune defense and to understand the molecular basis of octopus tolerance/resistance to coccidiosis.

**Results:**

A bi-directional mRNA library was constructed from hemocytes of two groups of octopus according to the infection by *A. octopiana*, sick octopus, suffering coccidiosis, and healthy octopus, and reads were *de novo* assembled together. The differential expression of transcripts was analysed using the general assembly as a reference for mapping the reads from each condition. After sequencing, a total of 75,571,280 high quality reads were obtained from the sick octopus group and 74,731,646 from the healthy group. The general transcriptome of the *O. vulgaris* hemocytes was assembled in 254,506 contigs. A total of 48,225 contigs were successfully identified, and 538 transcripts exhibited differential expression between groups of infection. The general transcriptome revealed genes involved in pathways like NF-kB, TLR and Complement. Differential expression of TLR-2, PGRP, C1q and PRDX genes due to infection was validated using RT-qPCR. In sick octopuses, only TLR-2 was up-regulated in hemocytes, but all of them were up-regulated in caecum and gills.

**Conclusion:**

The transcriptome reported here *de novo* establishes the first molecular clues to understand how the octopus immune system works and interacts with a highly pathogenic coccidian. The data provided here will contribute to identification of biomarkers for octopus resistance against pathogens, which could improve octopus farming in the near future.

## Introduction

*Octopus vulgaris* is the most important octopus species in worldwide fisheries [1], [2], and represents a major protein resource in most fish-eating countries. It is of great commercial importance in Mediterranean, South American and Asian countries as well as in the NW Atlantic coasts of Spain and Portugal [3]. However, in the last 10 years cephalopod fishery has increased due to the mollusc high price in the market, which has in turn favoured the development of octopus on-growing on an industrial scale [4]. The octopus on-growing is currently developed in tanks and in floating cages [4]–[6] with favourable results. However, high mortality has also been recorded [7]–[9] and as a result, several studies about the aetiology and prevention of diseases caused by different pathogens have been encouraged [10]–[12].

The gastrointestinal coccidian parasite *Aggregata octopiana* (Protozoa: Apicomplexa) has been noted as the most important epizootiological agent in wild and cultured octopus stocks from European waters [12], [13]. The infection by *A. octopiana* induces ulceration of the epithelium of caecum and intestine, partial destruction of the digestive tract, and decrease or malfunction of the absorption enzymes [10], [14]. Although enteric coccidiosis is not a primary cause of death, the induced malabsorption syndrome may impair octopus growth and health [10].

Hemocytes are the circulating cells of the hemolymph. They play a major role in processes like wound repair and nutrient transport, but are also important in the cellular defense against pathogens [15]. Although molluscs lack a specific immune system, the innate response mediated by circulating hemocytes and molecular effectors allows efficient and rapid responses to aggressors. The role of the hemolymph and the hemocytes in physiological functions and the immune system of bivalve molluscs have been the objective of a large amount of studies [15]–[19]. In contrast, only few studies related to cephalopod immuno-biology are available to date. Most of them report functional immune assays on the white octopus *Eledone cirrhosa* [20]–[22], the Pacific sepiola *Euprymna scolopes* (the only one detailing some molecular data) [23]–[27] and the common octopus, *O. vulgaris* [28]–[30].

Cephalopods are invertebrates showing innovative traits, such as no larval phase in ontogenesis, a vertebrate-like eye, a highly centralized nervous system and a close circulatory system, where the hemolymph is restricted to blood vessels and capillaries. All these characteristics indicate cephalopods as a highly evolved branch of molluscs, thus making them interesting models for neurobiological [31], learning [32], [33] and circulatory system studies [34]. However, molecular studies in cephalopods are at still at their beginning. In particular, the genome of *O. vulgaris,* has not yet been sequenced and no data exists about the molecular defense mechanisms underlying octopus-pathogen interactions. Nevertheless, a strategic plan aimed to promote the genome sequencing of different cephalopod species has recently been stated [35]. In the absence of genomic data, the high-throughput sequencing of total mRNA is a viable strategy for the study of the genes expressed in *Octopus vulgaris* [36]. Next Generation Sequencing, and Illumina short reads in particular, has successfully been used to build transcriptomic datasets in non-model species [37], [38]. The assembly of short reads data into contiguous sequences demonstrates that the assembly of long, potentially full-length transcripts assemblies is indeed possible [38].

Currently, transcriptomic studies on cephalopods have been restricted to an ecological framework in the sepiolid *E. scolopes* through cDNA libraries and EST collections [24], [39], [40]. The 454 pyrosequencing approach has been employed to understand the role of the circulating hemocytes of *E. scolopes* (colonized by the symbiotic bacteria *Vibrio fischeri)* in the squid/*Vibrio* association [41]. Only two transcriptomic studies have been performed to date related to the common octopus. The first one was an analysis of gene expression carried out through an EST collection of the *O. vulgaris* ocular chamber [42]. Recently, Illumina next generation sequencing technology was employed to characterize the transcriptome of the *O. vulgaris* central nervous system [43]. Due to its relatively low cost and good results obtained in octopus and other organisms, the Illumina RNA-Seq technology paired-end is a promising tool to study the octopus immune system as well.

In this study, we present the first hemocyte transcriptomic analysis of the cephalopod *O. vulgaris* by *de novo* sequencing and annotation of the data generated by high-throughput sequencing Illumina platform (GAII). The new data is expected to increase the publicly available sequence records of cephalopods substantially, especially considering genes involved in the cellular immune defensive activities of octopus hemocytes during coccidiosis.

## Results and Discussion

### Illumina sequencing and reads assembly

We used the paired-end Illumina sequencing platform to obtain the *de novo* transcriptome of the circulating hemocytes from adult octopus, and to analyse the octopus gene expression profile against infection by the parasite *A. octopiana*. Wild octopuses collected for the analysis were divided in two groups, one of five healthy octopuses harboring 0–2×10^3^ sporocyst per gram of digestive tissue tract (spor/g) and without digestive tissue damage, and a second group of five sick octopuses infected by 6×10^6^ to 2×10^7^ spor/g, showing inflammation, distention and necrosis of digestive tract tissue.

**Figure 1:**
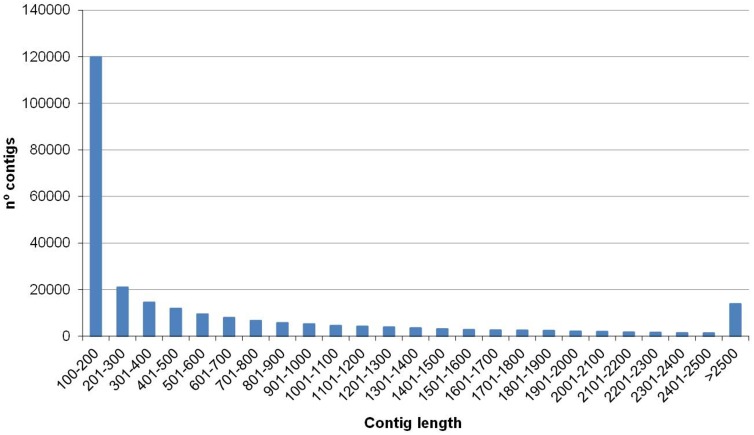
Length distribution of transcripts obtained from *O. vulgaris* hemocytes transcriptome library.

**Figure 2:**
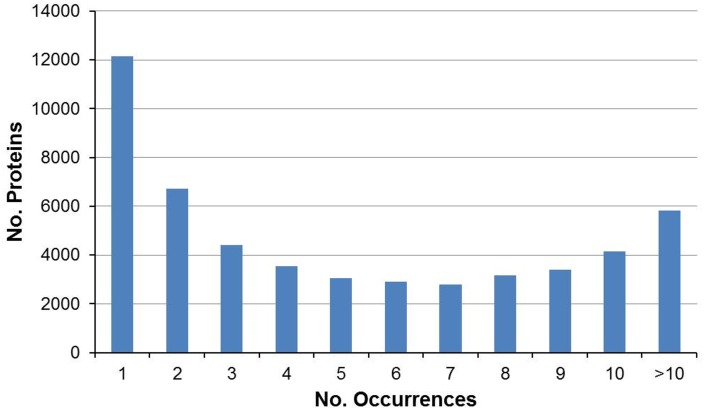
Frequency of contigs showing similarity to known proteins in the NCBI database.

A total of 150,302,926 reads (sequences) of 105 bp were generated by the Illumina sequencer. These reads correspond to the raw data of the experiment (Table1). The Q20 percentage (sequences of high quality indicator) was 97.6% (75,571,280 reads) for the pool of hemocytes from sick individuals (highly infected by the parasite *A. octopiana*, showing high parasite load) and 97% (74,731,646 reads) for the pool of healthy ones (showing null or low parasite load). After filtering to remove low quality reads, a total of 127,019,711 (84.5%) clean reads were obtained from both pooled hemocyte samples. Reads from both levels of infection were sequentially assembled together with Trinity [38] and Velvet [44]. In this manner, the transcriptome reflects specific transcripts from sick and healthy octopuses plus additional transcripts putatively common to both conditions. Further alignment of sequences belonging to both sick and healthy octopuses against the entire transcriptome allowed us to detect the transcript profile of each condition. Posteriorly, a comparative analysis of gene expression was performed between sick and healthy conditions. Through assembly, 254, 506 contigs (grouped in 228,314 clusters) with a mean length of 669 bp and a maximum of 19,120 bp were generated (Table 1). Hence, the theoretical transcriptome length for *O. vulgaris* was 170.24 Mb. Figure 1 shows the distribution of contig lengths. The frequency of contigs showing similarity to known proteins in the NCBI database is shown in Figure 2.

**Table 1:** Summary statistics of sequencing and assembly for *O. vulgaris* hemocytes transcriptome.

EST database summary	
**Sequences before Filtering**	
Number of reads	150,302,926
Total Megabases	15,781.8
**Sequences after Filtering**	
Number of reads	127,019,711
Total Megabases	13,180.8
**Assembly Statistics**	
Number of reads assembled	42,826,899
Number of contigs	254,506
Total consensus Megabases	170.24
Average contig length	669
N50 contig length	1,632
Range contig length	100–19,120
Number of contigs >500 bp	87,408
Number of clusters	228,314
Number of clusters with 1 contig	214,607
Number of clusters with >1 contig	13,707
Percentage of contigs annotated by SwissProt	18.9%
Percentage of contigs functionally annotated	13.7%

### BLASTx search in Swiss-Prot database

**Figure 3:**
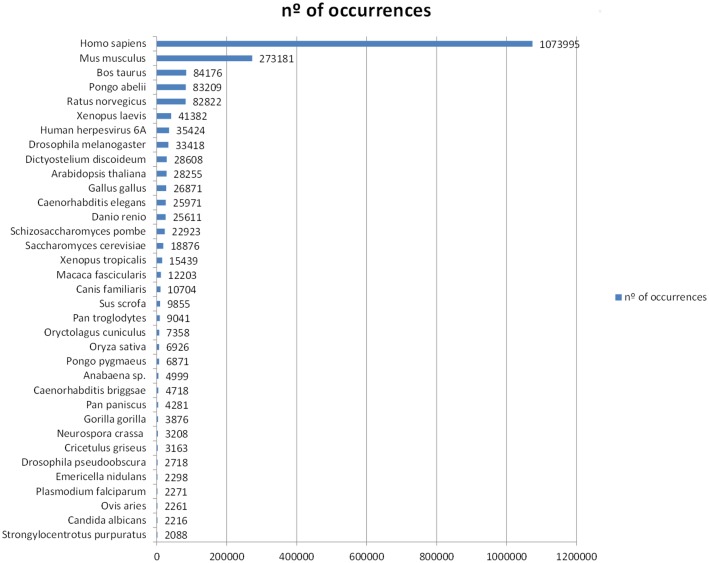
Top 35 hit sequences matching *O. vulgaris* assembled sequences.

Contig gene annotation was performed through BLASTx search against the SwissProt database using a cut-off e-value of 1e^−3^. Using this approach, a total of 48,225 (18.95%) contigs presented a significant BLASTx hit (e-value<1e^−3^) and were selected for annotation. The remaining, 81.05% of the assembled sequences did not match any known proteins probably because of the lack of molecular data of cephalopod species. Therefore, a high number of potentially novel genes could be included, but more genetic studies are needed to annotate them correctly. The species that were found with the most matching sequences was *Homo sapiens* (1,073,995 occurrences), whereas the sea urchin *Strongylocentrotus purpuratus* (with 2,088 occurrences at position 35) was the single marine invertebrate homologue to sequences of the common octopus in the top 35 species represented (Figure 3). Despite other marine mollusc species matched to our library, they were all below the top 35.

### Functional annotation based on GO

**Figure 4:**
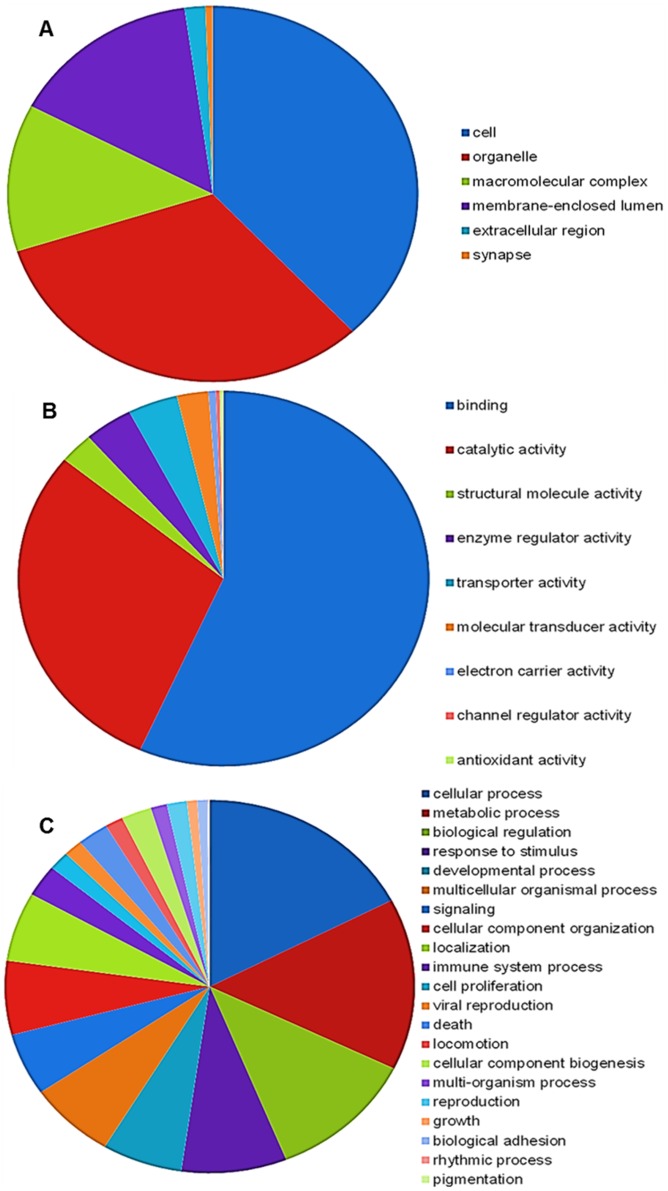
Distribution of second level GO annotation in three categories: (A) cellular component, (B) molecular function and (C) biological process.

Gene Ontology (GO) assignments were carried out at level 2 to classify the proteins putatively identified after blasting the Swissprot database based on sequences homology, into three main ontology categories: cellular component, molecular function and biological process. Relative to cellular components (Figure 4A), the highest percentage of GO corresponded to cell and organelle proteins, with 38% and 32% respectively. Within the molecular function category (Figure 4B), binding and catalytic activity were the most represented groups, with 57% and 29% respectively. Related to the biological process (Figure 4C) cellular (17%) and metabolic process (15%) were the highest represented groups; in addition, biological (12%) and response to stimulus (8%) also showed a high percentage of representative sequences.

### Comparative analysis of the *O. vulgaris* transcriptome

In order to understand similarities among the *O. vulgaris* transcriptome and their molluscan relatives, the general transcriptome of the hemocytes of *O. vulgaris* was compared to the bivalve species *Crassostrea gigas, Mytilus galloprovincialis and Ruditapes philippinarum,* which are well represented in public databases, and the cephalopod species *O. vulgaris* and *E. scolopes* which have been the subject of other studies. The transcripts obtained in this study matched 20% (6,402 hits) with the *O. vulgaris* sequences available in NCBI and 0.85% (301 hits) with those available for *E. scolopes*. The comparison with other mollusc species showed a match of 0.06% with *Crassostrea gigas* (135 hits), 0.40% with *Mytilus galloprovincialis* (79 hits) and 3.80% with *Ruditapes philippinarum* (900 hits).

These results clearly indicate the limited representation of molluscs, specifically cephalopods, in public databases. The mollusc sequences used for the comparative analysis were derived from different tissues, and only few of them come from hemocytes. In fact, for *O. vulgaris,* only 32,304 nucleotide sequences, 35 ESTs, 257 proteins and 13 genes are deposited in the GenBank databases to date. Most of these sequences are derived from taxonomic and central nervous system studies. Consequently, the results provided in this study highlight the need to increase the number of annotated sequences from cephalopods in public databases, which will help to discover new genes that would allow further understanding of the entire molluscan cephalopod biology. An additional file containing the largest contigs of each representative *locus* or gene selected for annotation is provided in Table S1.

### Immune transcriptome analysis

**Figure 5:**
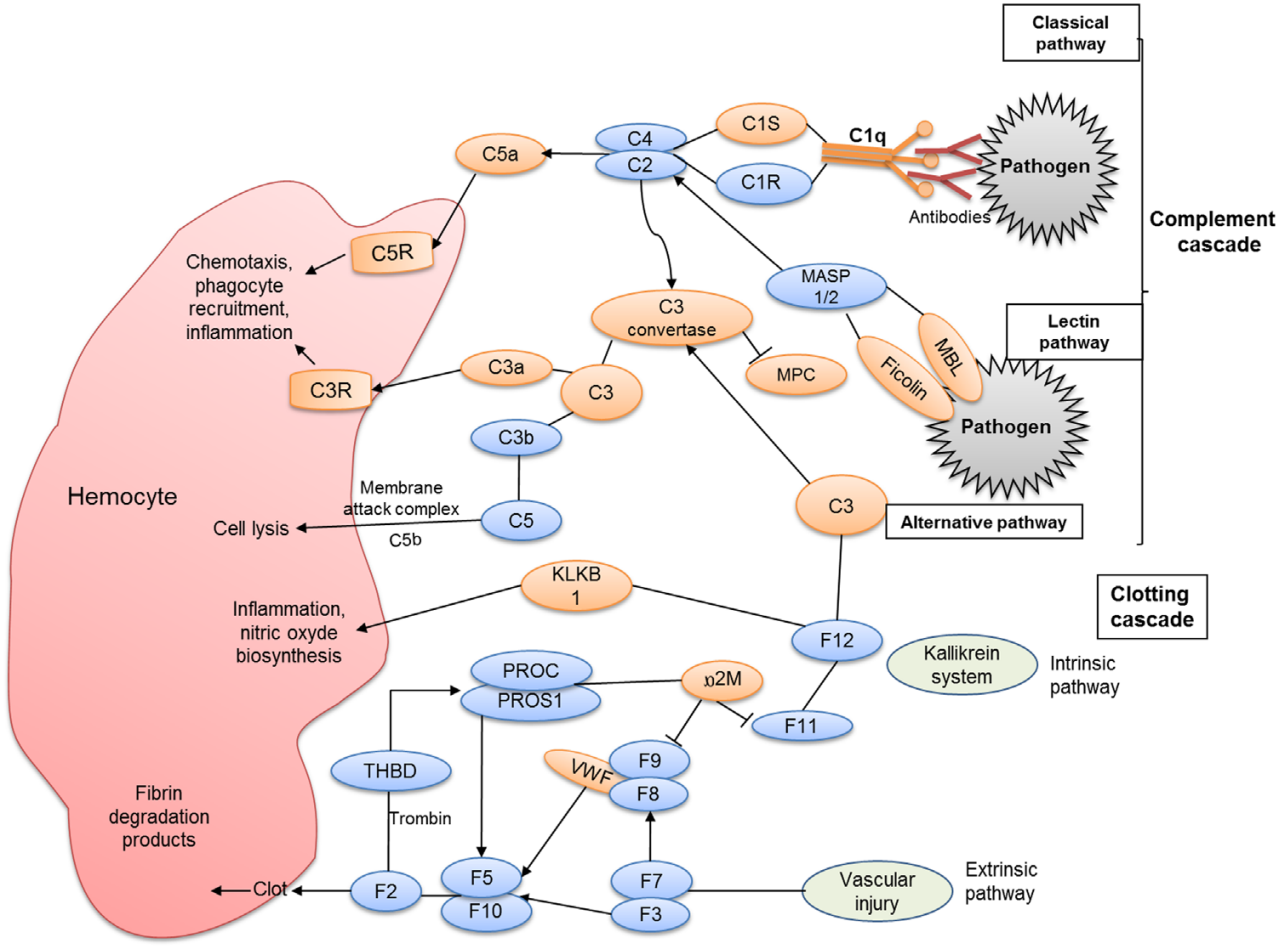
Complement and clotting pathway. Brown figures indicate proteins identified in the *O. vulgaris* library; and blue figures indicate the absent ones. C1q: Complement C1q binding protein; C1R: C1r subcomponent; C1S: Complement C1 subcomponent; C2: Complement component 2; C4: Complement component 4; C3: Complement component 3; C3a: anaphylatoxin subcomponent 3a; C3b: Opsonin subcomponent 3b; C5: Complement component; C3R: C3 receptor; C5R: C5 receptor; MBL: Manose-binding lectin; MASP1/2: Mannan-binding lectin serine protease 1/2; F12: factor 12; F11: Factor 11; α2M: Alpha-macroglobulin; F2,3,5,7,8,9,10: Coagulation factors 2,3,5,7,8,9,10; MPC: CD46, membrane cofactor protein; VWF: Von Willebrand factor; KLKB1: kallikrein B1; PROC: protein C; PROS1: protein S (alpha); THBD: trombomodulin.

**Figure 6:**
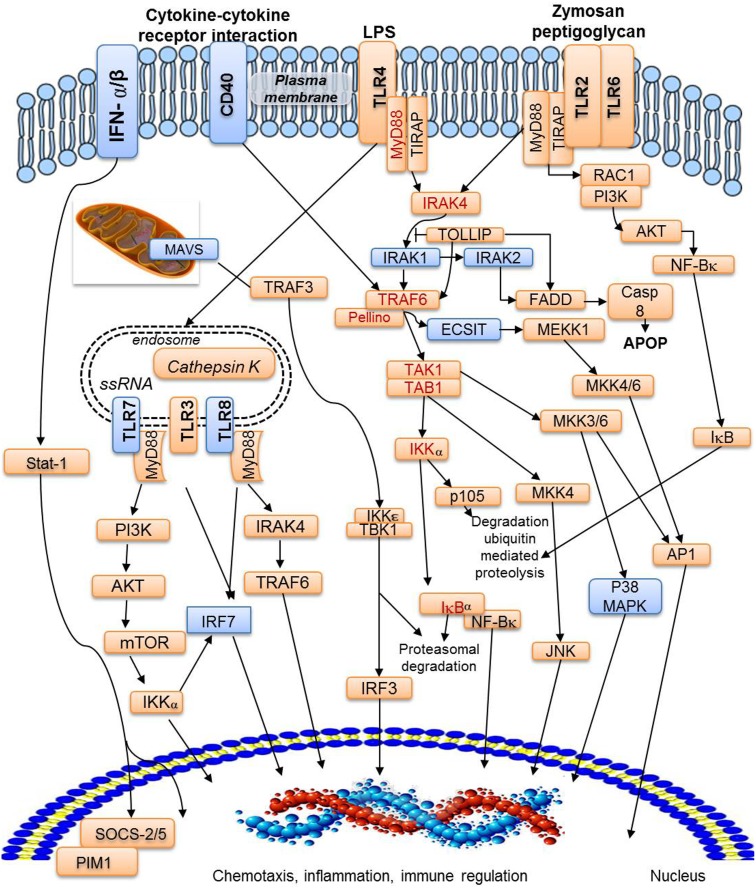
TLR/NF-κB signaling pathway. Brown rectangles indicate proteins identified in the present *Octopus vulgaris* library and blue rectangles indicate the absent ones. Brown rectangles with red letters indicate proteins in the NF-κB pathway. AKT: RAC-alpha serine/threonine-protein kinase; API1: Transcription factor AP-1; Casp8: Caspase 8; FADD: FAS-associated via death domain; IκB: Inhibitor of NF-κB; IKKε: Inhibitor of nuclear factor kappa-B kinase subunit epsilon; IRAK4: Interleukin-1 receptor-associated kinase 4; IRF3: Interferon regulatory factor 3; IκBα: NF-kappa-B inhibitor alpha; JNK: c-Jun N-terminal kinase; MEKK1: Mitogen-activated protein kinase knase 1; MKK4/6: Mitogen-activated protein kinase kinase 4/6; MyD88: Myeloid differentiation primary response protein 88; Mtor: Mechanistic target of rapamycin; NF-Κb: Nuclear factor kappa-B; PI3K: Phosphatidylinositol 3 kinase; PIM1: Proto-oncogene serine/threonine-protein kinase pim-1; p105: Nuclear factor NF-kappa-B p105 subunit; RAC1: Ras related C3 botulinum toxin substrate; Stat-1: Signal transducer and activator of transcription 1; SOCS-2/5: Suppressor of cytokine signaling; TAB1: TAK1-binding protein1; TAK1: TGF-beta activated protein kinase kinase 1; TIRAP: Toll-interleukin 1 receptor domain-containing adaptor protein; TLR2: Toll-like receptor 2; TLR4: Toll like receptor 4; TOLLIP: Toll interacting protein (├ direct inhibition); TRAF3: TNF receptor-associated factor 3; TRAF6: TNF receptor-associated factor 6; MAVS: Mitochondrial antiviral signaling protein that activates NF-kappa B and IRF 3; INFα/β: Interferon alpha/beta receptor; IRAK1–2: Interleukin receptor associated kinase 1, 2; IRF7: Interferon regulatory factor. P38MAPK: p38 mitogen-activated protein kinases; ECSIT: Evolutionarily conserved signaling intermediate in Toll pathways.

**Figure 7:**
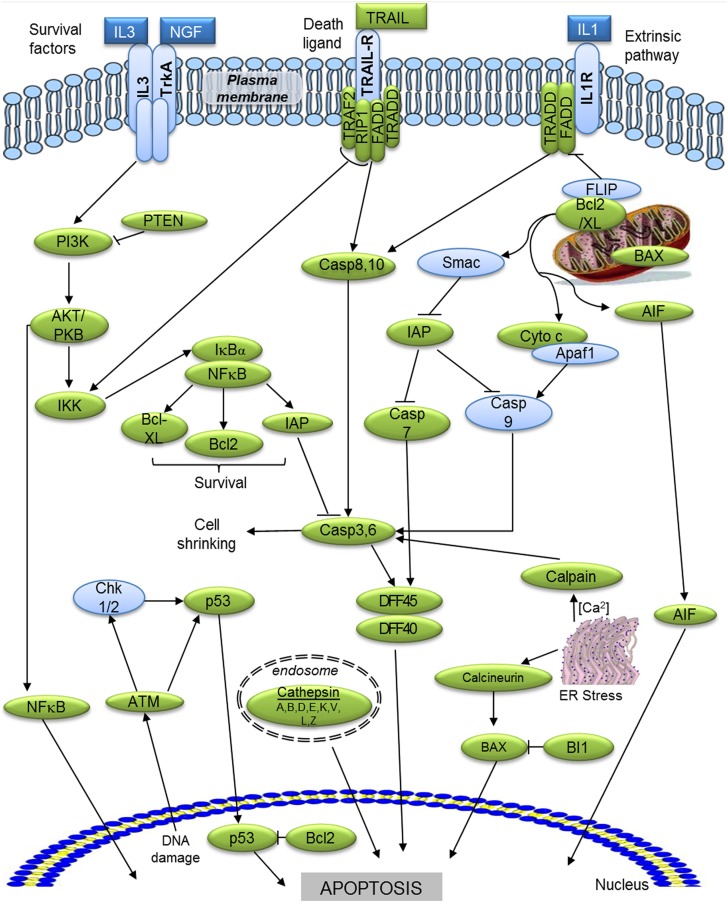
Apoptosis pathway. Green ellipse indicates proteins identified in the present *O. vulgaris* library and blue ones indicate absence. (├ direct inhibition). AKT/PKB: RAC-alpha serine/thereonine-protein kinse/Protein kinase B; AIF: Apoptosis-inducing factor 1 mitochondiral; ATM: Ataxia telangiectasia mutated protein; BAX: Apoptosis regulator BAX. Bcl2: Apoptosis regulator Bcl-2; Bcl-XL: Bcl-2 like protein 1; BI1: BAX inhibitor-1; Casp 3, 6, 7, 8, 10: Caspase 3, 6, 7, 8, 10; Cytc: Cytochrome C; DFF40, 45: DNA fragmentation factor of 40 kD, 45 kD; FADD: FAS-associated via death domain; IAP: Inhibitor of apoptosis; IKK: Inhibitor of nuclear factor kappa-Bkinase; IκBα: MyD88: Myeloid differentiation primary response protein MyD88; NF-kappa-B inhibitor alpha. IL3R: Interleukin 3 receptor; NFκB: Nuclear factor kappa-B; PI3K: Phosphatidylinositol 3-kinase; p53: Tumor suppressor p53. RIP1: Receptor interacting serine/threonine-protein kinase1; TRADD: TNF receptor superfamily 1 alpha-associated via death domain; TRAF2: TNF-receptor-associated factor 2; TRAIL: TNF-related apoptosis-inducing ligand; Apaf1: Apoptotic protease-activating factor; FLIP: FADD-like apoptosis regulator; PTEN: Phosphatidylinositol-3, 4, 5-trisphosphate 3 phosphatase and dual specificity protein phosphatase PTEN; Smac: Second mitochondria-derived activator of caspase; Chk1/2: Checkpoint kinases 1, 2.

Hemocytes are the key effectors of cellular defense activities against invading agents. When challenged by pathogens, the octopus raises a strong and effective innate immune response [45], [46] and therefore, immune genes are of particular interest to understand i) how the host-cell biological processes are altered by pathogens, specifically by the natural infection of the coccidia *A. octopiana*, and in consequence, ii) how the host immune system faces the infection. A selection of GO immune-related terms allowed us to identify more than 3% of the predicted proteins with a possible immune function. Among the different transcripts identified, a significant number of putative immune-related genes involved in several pathways like NFκB, TLR signalling pathway, complement cascade and apoptosis were recognized (Figure 5,6,7), suggesting that similar ancient mechanisms are shared with other molluscs.

#### 1. Complement pathway and related proteins

The complement system is a pathway comprised of more than 30 plasma and membrane-associated proteins that interact to the enhancement of cellular responses. Three different pathways activate complement: classical, lectin and alternative. All three have the component C3 in common, the central molecule where known activation pathways converge [47], [48]. Homologs to the complement C3 have been identified in marine organisms including the horseshoe crab *Carcinoscorpius rotundicauda* [49], the sea urchin *S. purpuratus* [50], the carpet-shell clam *Ruditapes decussatus* [51], the mussel *Mytilus galloprovincialis* [52] and the sea cucumber *Apostichopus japonicus* [53]. To date, the knowledge of the complement component C3 in cephalopods has been limited to identification and characterization in the sepiolid *E. scolopes* [39], [40], [54].

The *O. vulgaris* database presented herein contains putative homolog molecules of the complement signaling pathway (C3, C3R, C5R, C1S, MBL, Ficolin, C1q binding protein) (Figure 5). Additionally, highly important molecules including α_2_-Macroglobulin (3 transcripts) and fibronectin (2 transcripts) were also recorded in our *O. vulgaris* library (see Table S1).

#### 2. Pattern recognition receptors (PRRs)

**Lectins:** Lectins are sugar-specific binding proteins that take part in different roles such as in cell-to-cell interaction, signal transduction and protein folding, but they also take part in self/non-self-recognition [55], having a direct role in innate immune functions as LPS-binding molecules, agglutination, recognition and phagocytosis through opsonisation and complement-activating factors. Therefore, lectins are valuable to recognize potential invaders and may be critical to the internal defense of marine molluscs [56], [57]. Few reports regarding the isolation and biochemical characterization of lectins and their ability to recognise non-self molecules were identified in cephalopods. One lectin was biochemically characterized in *O. vulgaris* [58] and two others in *Octopus maya* [59], [60]. In the *O. vulgaris* library reported herein, homolog sequences of mannose binding C-lectin (MBL) (2 transcripts), galectin (1 transcript) and a different carbohydrate binding lectin (malectin) (1 transcript) have been putatively found.

**Peptidoglycan recognition proteins:** Peptidoglycan recognition proteins (PGRPs) specifically recognize bacterial peptidoglycan from Gram-positive and Gram-negative bacteria. This group of proteins is conserved from insects to mammals and has diverse functions in antimicrobial defense [61], [62]. To date, five PGRP transcripts with different characteristics and location are known in cephalopods, all of them identified in a cDNA library from the sepiolid *E. scolopes* [24], [41]. The analysis of the *O. vulgaris* library led to the identification of three PGRPs in the circulating hemocytes of the octopus for the first time. Further characterization of *O. vulgaris* PGRPs as well as studies to determinate their specific localization are required.

**Toll-like receptors:** Toll-like receptors are responsible for initiating inflammatory responses against invading pathogens in invertebrates and vertebrates. The Toll receptors provide the trans-membrane molecular link between the extracellular and intracellular compartments [63], [64]. Toll-like receptors and additional genes involved in this pathway have recently been described in *M. galloprovincialis* [65], [66] while in cephalopods several genes involved in this pathway were described in the light organ of *E. scolopes* [24]. The results obtained from our sequences showed transcripts encoding homologues to TLR-2, TLR-3, TLR-4 and TLR-6 (1 transcript respectively). In addition, most of the central proteins belonging to the TLR signalling pathway (the adaptor MyD88, IRAK and TRAF6 proteins) have also been identified in this transcriptomic analysis (Figure 6). Furthermore, several transcripts containing leucine rich repeat (LRR) domains and some immunoglobulin superfamily members also containing LRR have been identified in our library (48 transcripts).

#### 3. Cytokines

Cytokines are cell-signaling proteins that regulate inflammation and infection in the body [67]. They can be released through complement receptor-mediated signaling or by pathogens through a wide array of pattern recognition receptors (PRR) [68]. Two putatively identified transcripts of IL-17 were found in our *O. vulgaris* library. IL-17 is involved in the inflammatory process during infection and in the pathogenesis of chronic inflammation in autoimmune diseases. It is also capable of activating the NF-κB transcription factor in different cell types like macrophages or intestinal epithelial cells [69], [70]. In addition, the growth factors granulin (1 transcript), fibroblast growth factor 1 (FGF1) (1 transcript), fibroblast growth factor receptor 2 (FGRF2) (2 transcripts), transforming growth factor beta receptors (TGFβ) (2 transcripts), vascular endothelial growth factor (VEGF) (1 transcript), epidermal growth factor (EGF) (2 transcripts) and bone morphogenic protein (BMP) were found in the *O. vulgaris* transcriptome.

#### 4. NFκB pathway

The nuclear factor-κB (NF-κB) is rapidly activated by a wide group of agents and cellular stress conditions [71]. The NF-κB pathway seems to be an evolutionary conserved innate immune pathway that is also present in molluscs. Proteins of this pathway like Rel have been characterized in *C. gigas* [72] and *Haliotis diversicolor supertexta* hemocytes [73]. Likewise, IκB gene was characterized in the pearl oyster *P. fucata* [74] and recently, molecules like IKK, IκB and KKγ/NEMO were characterized in *M. galloprovincialis* [66]. In cephalopods, molecules belonging to the NF-κB pathway like IKKg, TRAF6 or IRAK4 were identified from juvenile *E. scolopes* light organs [24]. The transcripts found in this *O. vulgaris* library have a high similarity with the previous findings identified in *E. scolopes.* In addition, we have putatively identified molecules that have never before been reported in cephalopods including TRAF2, TRAF3, TRAF5, IKKα, IKKβ, RIP and TAK1 (Figure 6; Table S1).

#### 5. Antimicrobial peptides (AMPs)

Antimicrobial peptides are proteins with the broad ability to kill or neutralize Gram-negative and Gram-positive bacteria, fungi, parasites or viruses, interacting with and crossing cell envelope membranes by a multihit mechanism [75]. The bactericidal permeability-increasing protein (BPI) is an AMP produced by polymorphonuclear leukocytes, but also by epithelial cells. Mucosal epithelia that co-exist with microbes and microbial products expressing BPI probably contribute to the maintenance of immunologic homeostasis at mucosal surfaces [76]. At least three light-organ proteins in the BPI/LBP (lipopolysaccharide-binding protein) family have been sequenced from *E. scolopes* [77]. A single transcript of the BPI protein is provided in this *O. vulgaris* library. Additional studies are needed to understand the role of this protein in the octopus cellular defense.

#### 6. Stress response genes

Hemocytes are the primary line of defense against pathogens and one of the strategies to avoid infections is the release of reactive oxygen and nitrogen species to kill pathogens [17]. Related to cytotoxicity, one transcript of nitric oxide synthase (NOS) and nitric oxide synthase trafficker (NOSTRIN), respectively, were putatively identified in the *O. vulgaris* library. In addition, 3 transcripts homologous to superoxide dismutase (SOD), 1 transcript homologue to peroxiredoxins (Prxs), both involved in the antioxidant system [78], [79] were also recorded. Other redox factors such as peroxisome (3 transcripts) were also observed. Previous records found abundant transcripts of myeloperoxidase in the *E. scolopes* symbiotic light organ [80] as well as SOD, peroxirredoxins, peroxidases and glutathione peroxidase [41], [54].

Heat-shock proteins (HSPs) serve as molecular chaperones that protect cells from the toxic effects of heat and modulate the stress response [81], [82]. In addition, their activity is closely related to the innate immune response [83]. In the *O. vulgaris* library HSP13, HSP27, HSP70, HSP71, HSP74, HSP76, HSP83, HSP85 and HSP90 were putatively identified.

#### 7. Apoptosis

Apoptosis is a common physiological process to remove damaged or potentially dangerous cells, but it is also a major defense mechanism against pathogens [84]. The central components of the apoptosis pathway are the proteases caspases. Initiator caspases (caspase 2, 8, 9 and 10) cleave and activate the effector caspases (3, 6 and 7) [85]. Apoptosis has been studied in marine invertebrates such as the abalone *Haliotis diversicolor* [85], the mussel *M. galloprovincialis* [86]–[88] or the shrimp *Penaeus monodon* [89], but it has not been studied before in cephalopods. The analysis of the here reported *O. vulgaris* library led to the putative identification of two initiator caspases, namely caspase 8 (3 transcripts) and 10 (1 transcript); and three effector caspases, caspases 3 (4 transcripts), 6 (1 transcript) and 7 (4 transcript) (Figure 7).

#### 8. Other proteins

Serin protease inhibitor (SERPIN) proteins are important elements of the host defense to inactivate proteases secreted by pathogens and restrict their invasion [89], [90]. Protease inhibitors have been found in *Crassostrea virginica*, *C. gigas* [91], *Chlamys farreri* [92] and *Ruditapes philippinarum* [93], but have not been described in cephalopods. A total of 6 transcripts corresponding to SERPIN were putatively identified in the *O. vulgaris* library. Biochemical, functional and molecular characterization of SERPIN is needed to understand whether and how the octopus’ hemocytes use this protein to counteract coccidiosis.

Angiopoietin is a protein that regulates angiogenesis, the process of formation of new blood vessels from other pre-existent ones [94]. A protein putatively similar to angiopoietin-like 4 (2 transcripts) was identified in our *O. vulgaris* library, which is not surprising since cephalopods possess the most complex circulatory system of all invertebrates.

Peroxisome proliferator-activated receptors (PPARS) are, in general, anti-inflammatory and can interact with transcription factors involved in inflammation such as NF-κB, activator protein-1 (AP-1) and STAT [95]. A total of 3 transcripts corresponding to PPARS were found in the *O. vulgaris* library.

Cluster of differentiation (CDs) are cell surface molecules expressed on various cell types in the immune system. They have a defined structure that is recognized by a group of monoclonal antibodies and are used to associate cells with specific immune functions. Using this approach, certain CD markers have been revealed in small coelomocytes of the earthworm *Eisenia foetida* [96], [97], the purple sea urchin *Arbacia punctulata* [98], and the leech *Hirudo intestinalis* [99]. In cephalopods, the CD63 molecule (3 transcripts) was previously found in *E. scolopes* hemocytes [41]. In the present *O. vulgaris* library, sequences putatively similar to different CDs were recorded. However, further studies will be needed to characterize them.

LPS-induced TNF-α factor (LITAF) is a transcription factor that regulates inflammatory cytokines in response to LPS stimulation, and thus controls TNF-α expression. This gene has been identified in gastropods [100] and bivalves species [52], [93], [101]. In this study, we have found two transcripts similar to LITAF, which have not been reported in cephalopods before.

Allograft inflammatory factor-1 (AIF-1) is a cytokine-responsive macrophage molecule, inducible by such cytokines as IFN-g, IL1β or IL-18 [102]. AIF-1 has been characterized in the coelomocytes from the Antarctic sea urchin *Sterechinus neumayeri* [103] and the pearl oyster, *Pinctada martensii* [104]. In the present study, one transcript of AIF-1 is reported for the first time in cephalopods.

Results reported so far provide a general overview of the proteins putatively found encoded by the common octopus hemocytes. Cellular components and proteins involved in metabolic processes have been commonly found. However, the information provided here is mainly focused on the immune proteins expressed by the *O. vulgaris* hemocytes.

### Differentially expressed transcripts in response to coccidian infection

Tophat and Cufflinks programs were used to analyse the reads of both infection conditions and report differentially expressed transcripts using a rigorous statistical analysis. From the two sample groups, the assembled contigs were transformed into FPKM (Fragments per Kilo bases per Million reads) to calculate abundance differences of each gene with further false discovery rate analysis. Thus, a set of 538 transcripts was differentially expressed (*p<*0.05) between sick and healthy octopuses. Significant transcripts included molecules related to cell structure (actin, tubulin, filamin) and metabolism (NADH). However, transcripts related to immune system and involved in pathogen recognition (C1q, TLR, PGRP), apoptosis (BAX inhibitor) and antioxidant system (Peroxiredoxin PRDX), among others, were also recorded (Table S2). Of them, a total of 312 transcripts were successfully identified in public databases. The remaining 226 assembled sequences did not match to any known proteins, probably due to the scarcity of the molecular representation of cephalopod species.

### RT-qPCR of selected genes

**Figure 8:**
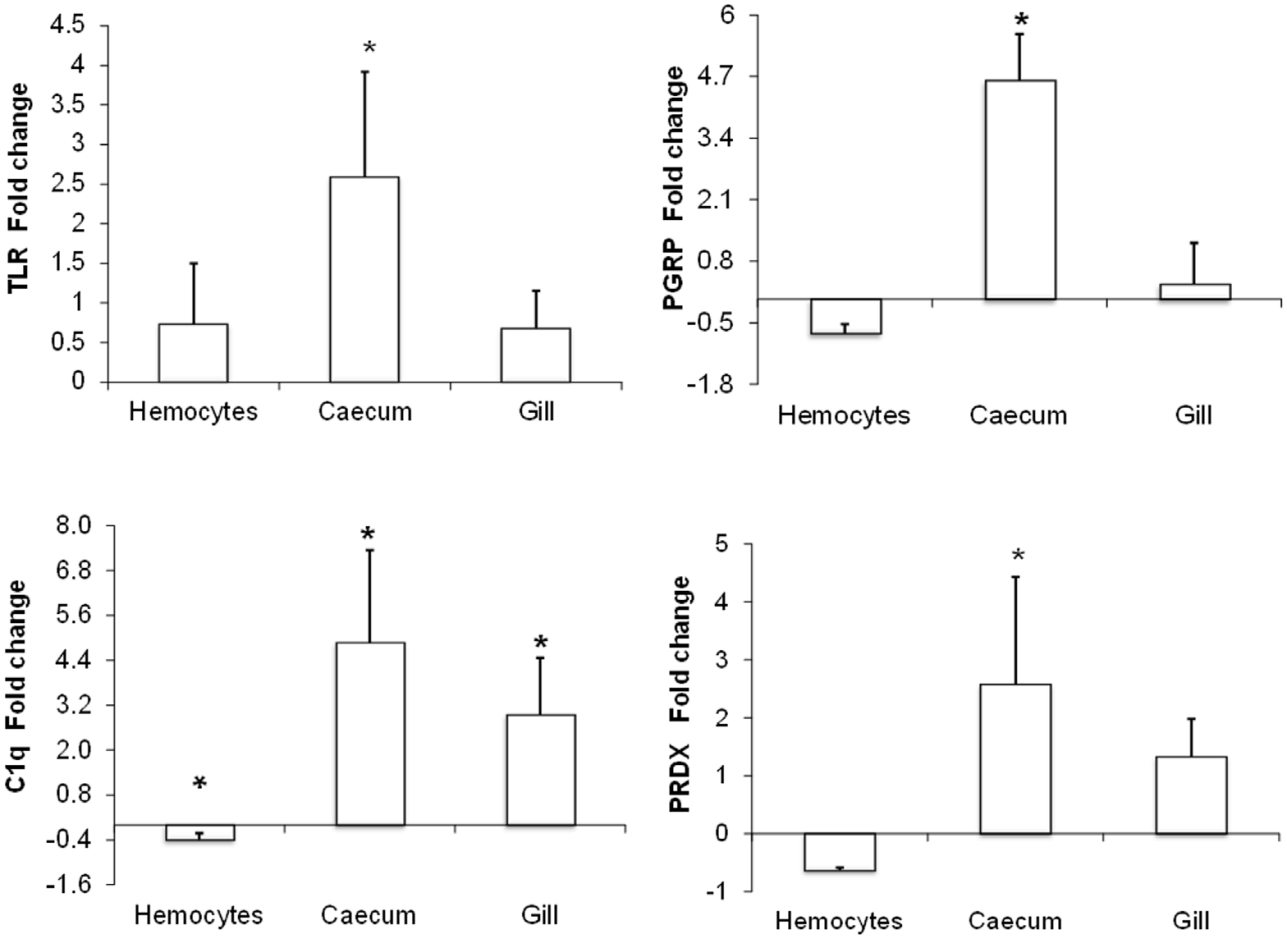
Fold change in gene expression analysis by RT-qPCR. Tissue expression profiles of immune genes in *O. vulgaris*. Data represent the fold change in expression of the analyzed transcripts relative to β-acting transcript level of sick octopuses (highly infected by *A. octopiana*), referred to healthy octopuses (null or lowly infected by *A. octopiana*). Results are mean ± standard deviation. Asterisks denotes significant differences (P<0.05).

To quantify differences in gene expression between sick and healthy octopuses, RT-qPCR was performed on genes such as PRRs (TLR, PGRP, C1q) and cell antioxidant system (PRDX) using specific primers (Table 2). Gene selection was based on gene implication in the host-immune response to pathogens and the differential expression observed in the transcriptomic library. Figure 8 demonstrated that the mRNA expression tested by RT-qPCR, followed the same trend of gene expression (in terms of up or down regulation pathway) as in the RNA-seq analysis. Consequently, RT-qPCR data supports the sequencing results and provides data about the suitability of using the Illumina sequencing approach for *de novo* assembly of the *O. vulgaris* hemocytes transcriptome without a genome reference.

**Table 2:** Primer sequences used for RT-qPCR.

Primer	Primer sequence 5′–3′	Amplicon Bp
**TLR F**	TATGGGTACCTGCAGATGGT	137
**TLR R**	TGAAAGCTGCTCATGTGAAA	
**PGRP-F**	GAGCTGCTCCACAACTGC	119
**PGRP-R**	CGACACCATTTCCACCA	
**C1q-F**	ACCAAGGTGGCACTGAGA	130
**C1q-R**	TCGCCCTCATGGAGAGT	
**PRDX-F**	CCAGTGCCAGTCTCTTTGAACA	100
**PRDX-R**	AGTGCACCTGGTACACCAAAAA	

In order to get an insight about the molecular effect of coccidiosis on the octopus immune defense, three different tissues were selected to perform the RT-qPCR analysis: hemocytes, responsible for the cellular defensive mechanisms [17], caecum, which is the target organ of the *A. octopiana* infection, and the gills, which are in permanent contact with the surrounding environment and potential pathogenic agents [105].

In hemocytes, only TLR-2 was up-regulated in sick octopuses (Figure 8), suggesting that these cells could detect parasite-derived ligands or endogenous molecules such as HSP and thus trigger inflammatory response [106]. From transcripts analyzed, only peroxiredoxin has been previously recorded weakly expressed at proteomic level in *O. vulgaris* hemocytes of octopuses highly infected by *A. octopiana* [107] providing evidence of the negative effect of severe coccidiosis in the octopus cellular immune defense.

Significantly high expression of PRRs and antioxidant genes was recorded in caecum of sick octopuses. TLR-2 has a crucial role in tolerance against commensal flora, recognizing pathogens and maintaining gastrointestinal homeostasis [108]. Likewise, PGRP also regulates the microbiota inside the gut [62]. High expression of TLR-2 (2.58 fold increase) is usually related to chronic inflammatory diseases, such as inflammatory bowel disease [109]. Thus, up-regulation of TLR-2 could be induced by tissue rupture and hemocytic infiltration originated by coccidiosis [14] in a disease specific manner similar to inflammatory bowel disease. Derived from high infection, microbiota is no longer controlled by PGRP and that could be the reason for such up-regulation (4.61 fold increase). However, an attempt to maintain homeostasis inside sick octopuses seems to be present. C1q is known to be produced in response to infection as inducers of pro-inflammatory activators [110] and seems to be activated in caecum of sick octopuses (4.86 fold increase) to induce proinflammatory response. At the same time, an additional cytotoxic defensive mechanism could be run to face coccidiosis and could be responsible for up-regulation (4.61 fold increase) of antioxidant proteins like PRDX to regulate the levels of toxic radicals that can also damage the host tissue [111].

From gills, C1q (2.93 fold increase) and PRDX (1.32 fold increase) were the highest up-regulated genes observed in sick octopuses. C1q could putatively work as an opsonising protein. Similar up-regulation was observed in protease inhibitors and PRDX, suggesting that the octopus cellular defense is acting against potential pathogens present in the seawater. The target organ of *A. octopiana* infection is the octopus digestive tract. Gills are not a target site of *A. octopiana*, but they can also be found infected [112], [113]. However, gills represent the main interface between aquatic organisms and the surrounding environment. Therefore, in molluscs, gills are not only valuable for oxygenation, but are also an important defense against infections [114], and thus the expression of the immune related genes in the octopus’ gills also has to be taken into account when studying immune response.

## Conclusion

The present study applied the high-throughput sequencing Illumina technology to provide the first data of the *O. vulgaris* immune system transcriptome. The successful results allowed the identification of a great number of new transcripts related to metabolic, functional and cellular components, but also transcripts of putatively new genes involved in the octopus immune response, which are herein provided for the first time. Sequences of molecules highly important for pathogen recognition and cellular homeostasis, belonging to pathways like complement, TLR and apoptosis were identified.

The inventory of the *O. vulgaris* genes involved in immunity evidenced that coccidiosis by *A. octopiana* induces differential expression profiles. Thus, the first insights of the effect of the *A. octopiana* infection at transcriptomic level, promoting the over-expression of some immune-related genes is herein provided. Finally, the information herein obtained would be useful i) to develop comparative immunology studies, ii) to better understanding of cephalopod’s immune response against pathogens, iii) and to characterize immune-relevant genes at the molecular and functional level in order to seek genetic markers of resistance that, in turn, will allow the development of future selective breeding programs to improve octopus farming.

## Materials and Methods

### Animal sampling, hemolymph extraction and selecting groups of infection

Specimens of *O. vulgaris* naturally infected by *A. octopiana* were collected by traps, an artisanal fishing gear used by local fishermen from the Ria of Vigo, Spain (24° 14.09′N, 8° 47.18′W). All the octopuses analyzed were sampled at the same locality, exposed to the same environmental conditions, showing similar length (DML: dorsal mantle length), and weighing around 1 kg (minimum legal weight of collection). Octopuses were maintained in tanks filled with filtered seawater at 15°C during 24 h. All experiments were carried out in accordance with the principles published in the European Animal directive (2010/63/EU) for the protection of experimental animals and approved by the Consejo Superior de Investigaciones Científicas (CSIC) ethics committee (Project number 10PXIB402116PR). Before hemolymph extraction, each octopus was anaesthetized using 7.5% magnesium chloride (MgCl_2_) according to Messenger [115], in strict accordance with ethical procedures and recommendations in order to minimize suffering [116], [117]. A dorsal incision was made through the skin and mantle muscle behind the head to withdraw hemolymph with a disposable syringe (1 ml) inserted directly into the cephalic aorta. One milliliter of hemolymph from each octopus was centrifuged at 12000×*g*, 4°C for 5 min. The pellet of hemocytes was re-suspended in 1 ml of Trizol reagent (Invitrogen) and stored at −80°C until the analysis was performed. The sacrifice was performed by immersion in frozen seawater (<1°C).

The digestive tract from each octopus was dissected and homogenized in 10 ml of filtered seawater (FSW) 1% Tween80 using an electric tissue grinder (IKA-Ultra Turrax T-25). After filtering, the number of sporocyst was counted in a Neubauer chamber. The sporocyst number is referred to as the number of parasites infecting a unit gram of octopus digestive tract (spor/g) in order to state the intensity of infection. The intensity of infection as well the histopathology produced was confirmed through the observation of caecum sections processed by standard histological methods [118]. Hence, taking into account both parameters (the intensity of infection and the histopathological damage), octopus were divided in two groups: the first one, showing a high parasite load, high intensity of infection (6×10^6^ to 2×10^7^ spor/g) and strong histological caecum damage; termed sick octopus group; and the second one, having a null or low parasite load, low intensity of infection (0 to 2×10^3^ spor/g), without histological caecum damage; termed healthy octopus group. The validity of both groups of infection were confirmed using a Student’s *t*-test analysis (*p<*0.05) over the sporocyst number date, performed in Statistica 6.0 software.

### RNA isolation, paired-end mRNA library preparation and sequencing

Total RNA from the hemocytes of 5 sick and 5 healthy octopuses selected from each group was extracted according to the Invitrogen protocol. After RNA extraction, samples were treated with Turbo DNase free (Ambion) to eliminate DNA. The RNA samples were purified using RNeasy Mini Kit (Qiagen), quantified using a NanoDrop ND1000 spectrophotometer and the RNA quality was assessed by Nano and Pico Chips Bioanalyzer (Agilent). A total of 1.5 µg of RNA from each of the 5 animals per group was pooled to construct the mRNA libraries according to the Illumina standard protocol. Thus, two mRNA libraries (one from the pool of sick octopus, and one from the pool of healthy octopus) were analyzed in a Genome Analyzer (GAII). In short, mRNA was purified using oligo (dT) probes and then fragmented into small pieces using divalent cations under a high temperature. The cleaved RNA fragments were used for first strand cDNA synthesis using random primers, modified and enriched for attachment to the Illumina flow cell. The two hemocyte libraries were generated using the mRNA sequencing sample preparation kit (Illumina). The libraries were validated by processing an Agilent DNA 1000 chip on a 2100 Bioanalyzer (Agilent) and quantified by qPCR using complementary primers of the library adapters with the KAPA SyBR FAST Universal qPCR kit (KAPA Biosystems). The cDNA libraries were sequenced on the Illumina sequencing platform (GAII equipped with a paired-end module) performing 105 cycles per read on two flow cell lanes.

The raw data are accessible in the NCBI Short Read Project (Accession number: SRP043705).

### *De novo* Transcriptome generation: transcript assembly

Prior to the assembly, filters to remove low quality reads and bases were applied using ConDeTri [119]. Base trimming was done from the 3′end of each read to remove bases with a quality less than Q20 up to a minimum length of 80 bases. Reads not reaching the 80 nucleotides in length were removed before further analysis. ConDeTri allows filtering in a paired manner. The filtered Illumina paired-end and remaining orphan reads from both sequenced samples were used together for assembly. First, an initial assembly was performed using Trinity [38]. The Trinity assembly was then used as a long sequence to guide re-assembly with Velvet [44]. The use of both software allowed us to test a wide range of K-mer lengths (25 for Trinity and 31, 35, 39, 43 for Velvet) and algorithms for assembly, and to obtain a consensus transcriptome that may cover the hemocyte transcriptome spectrum. Finally, Oases was used to produce a set of putative transcripts grouped in different genes or *loci* [120]. CD-HIT v4.5.4 [121], [122] was used to group similar transcripts into clusters. Two transcripts were grouped if at least 95% of the positions had at least 95% identity.

### Assembly validation and Functional annotation

To assess the coverage of the assembly, a homology search of the assembled transcriptome was performed against the Swissprot using BLASTx with an e-value threshold of 1e^−3^. BLASTx results were passed through a custom Perl script that merged the assembly Fasta sequence and summarized information to produce a table. Functional annotation was performed using Blast2GO v2.5.0 [123]–[125] with the default annotation parameters (Blast e-value threshold of 1e^−3^, Gene Ontology (GO) annotation threshold of 55). The GO terms associations for “Biological process”, “Molecular function” and “Cellular component” were performed using BLASTx algorithm against the Swissprot database.

### Comparative analysis

The library of the *O. vulgaris* hemocytes here generated was compared with sequences of the cephalopods *E. scolopes* (35,420 ESTs) and *O. vulgaris* (31,929 ESTs)*;* and the bivalves *M. galloprovincialis*, (19,617 ESTs), *C. gigas* (206,388 ESTs) and *R. philippinarum* (23,649 ESTs) deposited in the NCBI public database (accessed 5/6/2013). BLASTn algorithm was performed to test the sequence similarity with a threshold e-value less than 1e^−5^. The sequences were compared with the longest contig from each of the transcripts identified in *O. vulgaris* hemocytes.

### Identification of immune-related genes

To identify the putative genes involved in the immune response, the sequences obtained in this study were screened using the GO terms at level 2 assigned to each sequence after annotation and confirmation of its relationship with the immune response. They were also revised based on an immune system process and response to the stimulus keyword list elaborated in our lab. BLASTx was used to identify the putative immune related transcripts looking for these specific keywords in the hit descriptions of proteins of the NCBI database, which had shown to be involved in immune response. An important number of immune-related genes identified from our high-throughput sequencing results were grouped in 4 different pathways following the KEGG reference pathways [126], and related to: Complement system, Toll-like receptor, NF-κB and apoptosis.

### Transcripts differentially expressed against the infection

The differential expression of transcripts from sick and healthy animals was evaluated with TopHat [127] and Cufflinks [128] using the generated assembly as reference for mapping the reads from each condition and determining the relative transcript abundance by measuring FPKM (expected fragments per kilobase of transcript per million fragments). All *p-*values were adjusted with a false-discovery rate (FDR) correction for multiple testing according to the Benjamini-Hochberg method [129]. The transcripts were considered significant at *p<*0.05.

### Expression analysis of selected genes by quantitative real time PCR (RT-qPCR)

The differential expression of four genes selected from the transcriptome library and related to the innate immune response were analysed by RT-qPCR from three different tissues. Total RNA was extracted from the hemocytes, caecum and gills of 5 individual octopuses from each group (sick and healthy) using TRIZOL reagent (Invitrogen) and following the manufacture’s instruction. The RNA concentration was quantified using a NanoDrop ND2000 spectrophotometer (Thermo Scientific). First strand cDNA was synthesized using Maxima First Strand cDNA Synthesis Kit for RT-PCR (Thermo Scientific) using 1 µg of total RNA, treated with DNAse (QIAGEN) to remove the remaining genomic DNA. For each of the selected genes, forward and reverse primers were designed using primer 3 software (http://biotools.umassmed.edu/bioapps/primer3_www.cgi). PCR efficacy (*E*) was calculated for each primer pair by determining the slopes of standard curves according to Pfaffl [130]. The β-actin gene was determined as the best reference gene (HKG) through the NormFinder [131], geNorm [132] and Bestkeeeper [133] algorithms. RT-qPCR reactions were performed in triplicate with a total volume of 25 µl using a 7500 FAST Thermocycler (Applied Biosystems) sequence detector in 96-microwell plates. Each well contained 1 µl of cDNA (dilution 1/10), 12.5 µl of SYBR green PCR master mix (Thermo Scientific) and 0.5 µl of each diluted primer (10 µM). The standard cycling conditions were a two-step method: 95°C for 10 min and then 40 cycles of 95°C 15 s, and 60°C for 1 min. The expression of the selected genes was normalized using the β-actin gene and analysed following the Pfaffl method [130]. Results were expressed as the mean ± standard deviation. Fold units were calculated dividing the normalized expression values of tissues samples in sick individuals by the normalized expression values of healthy ones. Data were analyzed using a Student’s *t*-test and differences were considered statistically significant at *p<*0.05.

## Supporting Information

Table S1 **List of transcripts including the largest contig of each representative** ***locus*** **(e-values<1e^−3^) of** ***Octopus vulgaris*** **selected for annotation.**
(XLSX)

Table S2 **List of transcripts differentially expressed (P<0.05) between sick (S) and healthy (H) octopuses.** FC: Rate change. Expression rates of sick octopuses respect to the healthy. NA: Transcripts not identified in public databases. (#) denotes transcripts tested by RT-qPCR showed the same trend of gene expression as in the RNA-seq analysis, but without statistical significance (P>0.05).
(XLS)
